# Population projections for U.S. counties by age, sex, and race controlled to shared socioeconomic pathway

**DOI:** 10.1038/sdata.2019.5

**Published:** 2019-02-05

**Authors:** Mathew E. Hauer

**Affiliations:** 1 Department of Sociology, Florida State University, 600 W. College Avenue, Tallahassee, USA; 2 The Center for Demography and Population Health, Florida State University, Tallahassee, USA

**Keywords:** Sociology, Socioeconomic scenarios

## Abstract

Small area and subnational population projections are important for understanding long-term demographic changes. I provide county-level population projections by age, sex, and race in five-year intervals for the period 2020–2100 for all U.S. counties. Using historic U.S. census data in temporally rectified county boundaries and race groups for the period 1990–2015, I calculate cohort-change ratios (CCRs) and cohort-change differences (CCDs) for eighteen five-year age groups (0–85+ ), two sex groups (Male and Female), and four race groups (White NH, Black NH, Other NH, Hispanic) for all U.S counties. I then project these CCRs/CCDs using ARIMA models as inputs into Leslie matrix population projection models and control the projections to the Shared Socioeconomic Pathways. I validate the methods using ex-post facto evaluations using data from 1969–2000 to project 2000–2015. My results are reasonably accurate for this period. These data have numerous potential uses and can serve as inputs for addressing questions involving sub-national demographic change in the United States.

## Background & Summary

Population projections have a long history in the social and physical sciences as a means of examining demographic change, planning for the future, and to inform decision making in a variety of applications^1–7^. Scholars typically produce detailed population projections for countries^6,8^, but growing demand for small-area demographic analysis, especially as it relates to climate change, highlights the importance of subnational projections^9–14^.

Despite the growing demand for subnational population projections, relatively few subnational population projections in the United States exist. County-level population projections are typically only available through the gray-literature (such as through the Federal and State Cooperative for Population Projections) or through for-profit companies and oftentimes only comprise several states rather than the whole United States. These projections, while incredibly useful, tend to employ a variety of methods, input data, time horizons, and demographic groupings making inter-state and inter-projection comparisons difficult. Other research has turned to gridded-population projections for subnational analysis^14^. Such data are useful, but lack demographic details by age, sex, or race and utilize geographies uncommon to other United States statistical reporting. The lack of rigorous small-area population projections by detailed demographic subgroups has likely hampered our understanding of subnational demographic change in the United States.

The Cohort-component method for population projection, the typical demographic projection methodology, requires oftentimes difficult-to-obtain (if not impossible) data on each population component process (fertility, mortality, and migration), and this data limitation generally limits population projections to the nation scale where such data are commonly available^6,8^. Using a parsimonious cohort-component alternative^15^, I overcome the data issues associated with a typical cohort-component projection to produce a set of U.S. county-level population projections by detailed demographic characteristics (eighteen age groups, two sex groups, and four race groups) controlled to the five Shared Socioeconomic Pathways (SSPs)^8^ and make both the *R* code and subsequent population projections available for dissemination to a wide audience. These projections can be used to understand small-area demographic change in the United States.

The Hamilton-Perry method^16,17^ is a simple, parsimonious technique for producing population projections directly from multiple age-sex distributions using cohort-change ratios (CCRs)^15^ andis a common alternative to cohort-component. The minimal data requirements to produce CCRs and the ability to implement CCRs in Leslie matrix projection methods^18^ make CCRs attractive in the production of small-area demographic projections. However, CCRs suffer from two major disadvantages over the use of cohort-component: 1) short-term rapid population growth can create impossibly explosive growth in long-range projections due to the nature of compound growth and 2) small cell sizes can create impossibly large CCRs with very small numeric change (ie 2 persons –>4 persons yield a doubling each period).

I use an alternative to CCRs, which I call cohort-change differences (CCDs), which create linear rather than exponential growth in a blended model where county-race groups projected to grow utilize CCDs while county-race groups projected to decline utilize CCRs. Blended linear/exponential demographic projections tend to outperform both linear and exponential models, respectively^19^. This technique has all of the advantages of CCRs by remaining just as simple and parsimonious with minimal data requirements while producing projected populations without impossibly explosive growth. I use autoregressive integrated moving average (ARIMA) models to project the CCRs/CCDs. All individual CCRs/CCDs (*CCR*_*asrc*_) over all series are modeled (n = 336024) in individual ARIMA models that populate the Leslie matrices for projection. I then control the resultant projected age-sex structures to the five SSPs^8^.

Out-of-sample validation reveals errors on par with or better than cohort-component population projection models undertaken at the national and sub-national scale^18–22^.

### Methods

The cohort-component method is the most accepted methodology to produce population projections^1,23^. The method makes use of all three population component processes (fertility, mortality, and migration) and applies them across varying population cohorts to arrive at a future population. Equation 1 outlines the basic structure of a cohort-component model.

(1)Pt+1=Pt+Bt−Dt+Mt,in−Mt,out

Where *P*_*t*_ is the population at time *t*, *B*_*t*_ is the births at time *t*, *D*_*t*_ is the deaths at time *t*, and *M*_*t*,*in/out*_ refers to in- or out-migration at time *t*.

Cohort-component requires data on each component process disaggregated by the dimensionality of the population to be projected. To produce detailed projections by age, sex, and race, detailed data by age, sex, and race for each component of change must be available. Certain elements of the components of change data can be difficult to obtain for complete national coverage of sub-national geographies. For example, there is no comprehensive data set of both in- and out-migration estimates by age, sex, and race for all U.S. counties. Birth and death data are typically obtained through the National Center for Health Statistics (NCHS) vital events registration databases^24^. Birth data, however, are only available for counties with populations greater than 100 k and Death data are only available for cells with more than10 deaths^25^. These limitations surrounding fertility, mortality, and migration render a universalcounty-level population projection difficult, if not impossible, to complete using publicly available data sets using a traditional cohort-component model.

An alternative to cohort-component is the Hamilton-Perry method^15,17^, which uses cohort-change ratios (CCRs) in place of components to project populations. The general form of the CCR equation is found in Equation 2.

(2)CCRx,t=nPx,tnPx−y,t−y

(3)nPˆx,t+y=CCRx,t⋅nPx−y,t

Where _*n*_*P*_*x*,*t*_ is the population aged *x* to *x* + *n* in time *t* and _*n*_*P*_*x*-*y*,*t*_ is the population aged *x* − *y* to *x *+* n* − *y* in time *t* where *y* refers to the time difference between time periods. These CCRs are calculated for each age group *a*, for each sex group *s*, for each race group *r*, in each time period *t*, in each county *c*. Thus to find the population of ten to fourteen year olds (_5_P_10_) in five years (*t* + 5), we multiply the ratio of the population aged 10–14 in time *t* (_5_*P*_10,*t*_) to the population aged 5–9 five-years prior in time *t*-5 (_5_*P*_5,*t*-5_) to the population aged 5–9 in time *t* (_5_*P*_5,*t*_). ie, if we have 100 5–9 year olds five years ago and we now have 125 10–14 year olds and 90 5–9 year olds, we can project the number of 10–14 year olds in 5 years to be (125/100∙90 = 112.5).

CCRs offer several advantages and disadvantages over the use of a cohort-component model. CCRs are considerably more parsimonious than cohort-component. Calculation of CCRs for use in population projections requires data as minimal as an age-sex distributions at two time periods – data ubiquitous across multiple scales, countries, and time periods. However, this parsimony comes at a relatively steep price: CCRs can lead to impossibly explosive growth in 1) long-range projections due to the natural compounding of the ratios and 2) in small cell sizes with impossibly large CCRs due to a small numeric change in population. Consider the growth presently occurring in McKenzie County, North Dakota (FIPS code = 38053) driven by the shale oil boom. In 2010 McKenzie had a population of 6,360 that had ballooned to 12,792 by 2015, according to the Vintage 2016 population estimates from the US Census Bureau, with a CCR for the 20–24 year old population of 2.46 (416 to 1,027 persons). Implementing a50-year population projection using that CCR would create a projected population that is approximately 8,000 times larger (2.46^10^) – clearly an improbable number given the small, rural nature of itspopulation – yielding a potential population of approximately 8,000,000. As another example, Loving County, Texas (FIPS code = 48301) has 2017 estimated population of just 134 persons. Large numeric change in any given age group could lead to impossibly large CCRs in a county as sparsely populated as Loving County.

### Cohort Change Differences

The implementation of CCRs naturally implies a multiplicative model, typically utilizing Leslie matrices. It is possible, however, to implement an **additive** model by using the *difference* in populations rather than the *ratio* of populations.

(4)CCDx,t=nPx,t−nPx−y,t−ynPˆx,t+y=CCDx,t+nPx−y,t

**Figure 1:**
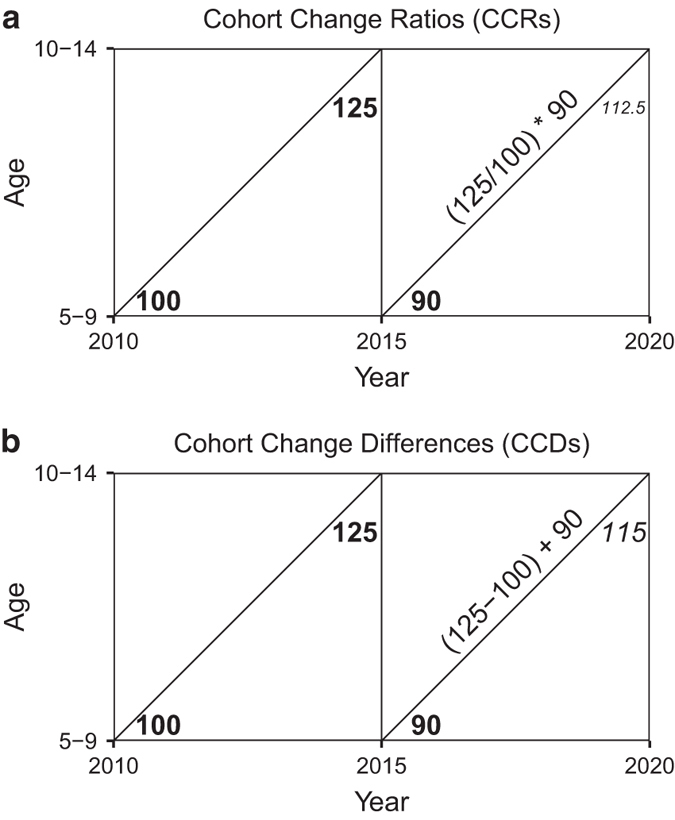
Lexis Diagrams for CCRs and CCDs. (**a**) Demonstrates the general framework for Cohort-change ratios and (**b**) the general framework for cohort-change differences using a toy example. The “observed” populations are in bold while the projected populations are italicized.

Thus to project the population of ten to fourteen year olds (_5_*P*_10_) in five years (*t* − 5), we take the difference between the population aged 10–14 in time *t* (_5_*P*_10,*t*_) and the population aged 5–9 five-years prior in time *t*-5 (_5_*P*_5,*t*-5_), and add this difference to the population aged 5–9 in time *t* (_5_*P*_5,*t*_). ie, if we have 100 5–9 year olds five years ago and we now have 125 10–14 year olds and 90 5–9 year olds, we project the number of 10–14 year olds in 5 years to be (125 − 100 + 90 = 115). Figure 1 demonstrates the similarities of using CCRs and CCDs in a lexis diagram.

CCDs are just as parsimonious as CCRs but have the additional advantage of producing linear growth rather than exponential growth. Using the same example as McKenzie County, ND, a numeric change of 611 persons in the 20–24 year age group (416 to 1,027) yields a potential population change of approximately just 6,000 persons over 50 years rather than 8,000,000 (when using a CCR) – much more realistic growth. However, for areas experiencing population declines, CCDs have the potential of creating impossible negative populations through linear decline. In this work, I use a blended approach in which CCDs in areas projected to increase and CCRs in areas projected to decrease create more utility in the projections, limiting impossible negative populations and explosive population growth, and previous research has shown blended linear/exponential population projections outperform both linear and exponential models, respectively^19^.

### Projecting CCRs and CCDs

To project the CCRs/CCDs, I employ the use of an autoregressive integrated moving average (ARIMA) model for forecasting equally spaced univariate time series data. I use an ARIMA(0, 1, 1) model which produces forecasts equivalent to simple exponential smoothing. All projections were undertaken in **R**^26^ using the forecast package^27^.

Where an ARIMA(0, 1, 1) model is

(5)Yt=Yt−1+et−θet−1

(6)Yˆt+1=Yt−θet−1

where *e*_*t*_ is independent and identically distributed as $N(0,\sigma_{e}^{2})$. It can be shown that ${\hat{Y}}_{t+1}$ is an exponentially weighted moving average of the observations $Y_{t},Y_{t-1},...$ with weights $\theta_{1}^{k}(1-\theta_{1})k=0,1,...,$ and that the additional forecasts ${\hat{Y}}_{t+j}$ for *j* > 1 remain constant at ${\hat{Y}}_{t+1}$^28^, [p.158].

I model all individual CCRs/CCDs (*CCR*_*asrc*_) over all series (n = 336024) in individual ARIMA models. I then input the projected CCRs and CCDs into Leslie matrices to create projected populations^29^.

There must be special consideration for two specific age groups: the populations aged 0–4 (_5_*P*_0_) and the population comprising the open-ended interval (_∞_*P*_85_). The populations aged 0–4 (_5_*P*_0_) and 85+ (_∞_*P*_85_) must have special consideration since the preceding/proceeding age groups do not exist for these age groups.

To project 0–4 year olds, I use the child-woman ratio (CWR)

(7)CWRt=5P0,t35W15,t5Pˆ0,t+y=CWR^t+y⋅35Wˆ15,t+y

Where ${\;}_{35}{\hat{W}}_{15,t+y}$ is the projected population of women in childbearing ages 15–49 at time *t* + *y*. I use the state/race-specific CWRs for member counties.

The population aged 0–4 in time *t* + 5 are projected by assuming a 1.05 sex ratio at birth (SRB) for the projected children born of women of childbearing age [15, 50), in time *t* + 5.

To calculate the CCD/CCR for the open-ended age group,

(8)CCR85,t=∞P85,t∞P80,t−y∞Pˆ85,t+y=CCR^85,t+y⋅∞P80,t

(9)CCD85,t=∞P85,t−∞P80,t−y∞Pˆ85,t+y=CCD^85,t+y+∞P80,t

CCR^x,t+y, CCD^x,t+y, and CWR^t+y refer to the projected values obtained from the individual ARIMA models (Equation 5).

If a given race/county combination is projected to increase, I use CCDs and if a given race/county combination is projected to decline, I use CCRs.

### Group quarters

The Group Quarters (GQ) population is a relatively small % of the US total population (just 2.6% of the US population resided in GQ in Census 2010) but still requires extra consideration. Prisons, college dormitories, nursing homes, and military barracks are some examples of GQ. I also include those without permanent living facilities (i.e., the homeless population) in my estimate of GQ. Unlike the resident population, the typical demographic structure of a GQ oftentimes remains constant and the underlying populations lack exposure to typical demographic processes in the same manner as the resident population. College dormitory populations do not age, are nearly always between the ages of 18 and 22, and fertility rates among college students are very low, for instance. Rather than demographic processes that change GQ populations, change is often the result of local, state, and federal policymaking resulting in a new prison, military base realignment, a new college dormitory, etc. These structural changes are difficult to predict without detailed knowledge of local decision-making. For this reason, I hold GQ constant throughout the projection horizon.

I calculate GQ as the difference between the household population and the total population in each age/sex/race/county group from Summary File 1 of the 2000 Decennial Census for the out-of-sample validation and from Summary File 1 of the 2010 Decennial Census for the population projections. This difference is the Group quarters population.

I project the household population using my methodology where the *household* populations are projected such that the populations at launch year are equal to the total population minus the group quarters population. Group quarters populations at time *t* are then added back into the projected household population to obtain the projected resident populations at time *t* + 5. This effectively projects the GQ population of each county as constant at its base value.

### Data

Data used to project the populations consist of a single primary data source: the National Vital Statistics System (NVSS) U.S. Census Populations with Bridged Race Categories data set (https://seer.cancer.gov/popdata/download.html). These data harmonize racial classifications across disparate time periods to allow population estimates to be sufficiently comparable across space and time. All county boundaries are generally rectified as well. The National Center for Health Statistics bridge the 31 race categories used in Census 2000 and 2010 with the four race categories used in the 1977 Office of Management and Budget standards.

There are two primary bridged-race data sets. The first covers the period 1969–2016 and utilizes three race groups: White, Black, and Other. The second covers the period 1990–2016 and uses four race groups (White, Black, American Indian/Alaska Native, and Asian/Pacific Islander) as well as two origin groups (Hispanic and Non-Hispanic). Due to small cell sizes, I convert the eight possible race classifications in the 1990–2016 bridged-race data to just four race groups (White NH, Black NH, Hispanic, and Other NH). Out-of-sample validation makes use of the three-race-group data set covering 1969–2016 while the actual population projections use the 1990–2016 data.

In the **Technical Validation**, I only consider counties that existed prior to year 2000 and are contained in the NVSS data. NVSS aggregated all counties in Hawaii to the state-level in the 1969–2016 NVSS bridged race data and I exclude them from the out-of-sample validation. Several counties were created after 2000 (most notably is Broomfield County, Colorado). The 15 counties excluded from the technical validation due to boundary changes or other reasons are Hoonah-Angoon Census Area AK 02105, Kusilvak Census Area AK 02158, Prince of Wales-Outer Ketchikan Census Area AK 02201, Skagway-Hoonah-Angoon Census Area AK 02232, Wrangell-Petersburg Census Area AK 02280, Adams County CO 08001, Boulder County CO 08013, Broomfield County CO 08014, Jefferson County CO 08059, Weld County CO 08123, Hawaii County HI 15001, Honolulu County HI 15003, Kalawao County HI 15005, Kauai County HI 15007, and Maui County HI 15009.

### Projection Controls

As shown below, any set of population projections using these methods are likely to produce higher than expected populations (see Tables 1, 2, and Supplementary Figure 1). To prevent runaway population growth, I control the projected output to the Shared Socioeconomic Pathways (SSPs)^8^. The SSPs are socio-economic scenarios that derive emissions scenarios coupled with climate policies. They are designed to evaluate both climate change impacts and adaptation measures in harmony with the Representative Concentration Pathways (RCPs) for emission scenarios. Scholars have downscaled the SSPs to incredibly detailed gridded population projections^14^, but they lack detailed demographic characteristics.

**Figure 2:**
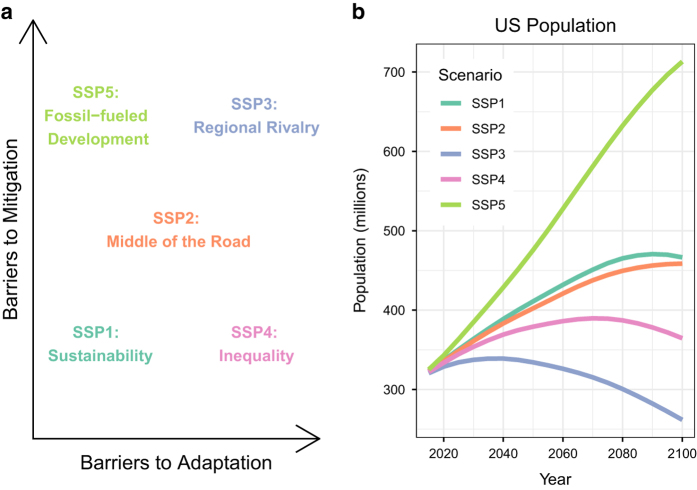
The five Shared Socioeconomic Pathways (SSPs). Adapted from^29^. (**a**) Shows the relationship between mitigation and adaptation and the five SSPs while (**b**) shows the projected populations under the five SSPs.

The five SSPs are colloquially named SSP1 (Sustainability), SSP2 (Middle of the Road), SSP3 (Regional Rivalry), SSP4 (Inequality), and SSP5 (Fossil-fueled Development)^30^. These five SSPs cover potential futures involving various growth policies, fossil-fuel usage, mitigation policies (ie emission reductions), adaptation policies (ie deployment of flood defenses), and population change^31^. Figure 2 shows the five SSPs and their relationship to barriers to mitigation (along the vertical axis) and barriers to adaptation (along the horizontal axis) and the associated projected US population for the scenarios. SSP1 (Sustainability) describes a future with low barriers to both mitigation and adaptation. Conversely, SSP3 (Regional Rivalry) describes a future with high barriers to both mitigation and adaptation.

Ultimately, SSP1 and SSP5 envision a future with optimistic human development but SSP1 contains a shift toward sustainability and SSP5 contains a continued fossil fuel-based, energy-intensive future. The difference in drivers leads to a medium projected population under SSP1 but a very high projected population under SSP5. SSP3 and SSP4 represent less educational investment and health, leading to increasing inequality. Population growth is low in industrialized countries under SSP3 and medium-low under SSP4^32^.

Each SSP contains projected population information in five-year increments for 5-year age groups (0–100+) and two sex groups (Male and Female) for the period 2020–2100 and I truncate the open-ended interval from 100+ to 85+ to be consistent with NVSS population estimates. I control my projected age/sex/race/county projections to the SSPs by using

(10)Pt=pasrc,tpas,t⋅Pas,SSP,t

where *p*_*asrc*,*t*_ refers to the age/sex/race/county specific population projected as outlined above at projected time *t*, *p*_*as*,*t*_ refers to the age/sex specific population projection at time *t*, and *P*_*as*,*SSP*,*t*_ refers to the age/sex specific population projection for each SSP at time *t*. This control allows preservation of the underlying age structures, race projections, and sex ratios, while ensuring the populations total the SSPs.

I only introduce the SSPs to control the projections for 2020–2100. The technical validation does not use the SSPs as controls.

### Code availability

All *R* code used to reproduce this analysis are available at https://github.com/mathewhauer/county_projections_official.

## Data Records

The projected populations by age/sex/race/county/year/SSP for all US counties for the period 2020–2100 are available at the Open Science Foundation (https://dx.doi.org/10.17605/OSF.IO/9YNFC).

Data resulting from these projections can be found in SSP_asrc.zip (Data Citation 1).

Projected populations include each US county, 18 age groups (1 = 0–4, 2 = 5-9, …, 18 = 85+), two sex groups (1 = Male and 2 = Female), and four race groups (1 = White NH, 2 = Black NH, 3 = Hispanic, and 4 = Other NH).

## Technical Validation

To evaluate the projection accuracy, I use the base period 1969–2000 to project the population for eighteen age groups, two sexes, three races (White, Black, Other), and 3127 counties for the projection period 2000–2015. I utilize an ex-post facto analysis at periods 2005, 2010, and 2015 using a pure CCD model, a pure CCR model, and blended model (CCD/CCR). The CCD/CCR model utilizes CCDs ifa county is projected to grow and CCRs if it is projected to decline. Blended models have been shownto outperform both purely linear or purely exponential models in simple extrapolation approaches to population projections^19^.

In keeping with demographic tradition^1,20,33^, I evaluate the projections using three primary statistics. To determine the overall accuracy of the projections, I use Absolute Percent Errors (APE) and to determine the bias of the projections I use the Algebraic Percent Error (ALPE). In the overall joint evaluations (age/sex/race/county) I have substituted the Symmetric Absolute Percent Error (SAPE)to account for possible zero cells^34^.

Equations 12 – 13 describe the equations used to evaluate errors. *P*_*i*_ refers to the projected value and *A*_*i*_ refers to the actual, observed value.

(11)APE=|Ai−PiAi|

(12)ALPE=Ai−PiAi

(13)SAPE=|(Pi−Ai)|(Pi+Ai)

### Age, Sex, Race, County joint errors

Table 1 shows the joint errors associated with all possible Age/Sex/Race/County combinations. Here the median error for any given ASRC combination (such as Black Females aged 20–24 in Lincoln County NV) is approximately 11–13% for all three methods after 15 years. These errors are on par with or better than many cohort-component models, as shown later.

### Overall Errors

Table 2 reports the overall errors for the sum of the population for the whole US. Overall the pure CCD model outperformed the purely CCR model, suggesting CCDs in this model could produce more accurate results compared to CCRs. All model variants (CCD, CCR, and CCD/CCR) tend to over-project the overall population in the United States.

Table 3 reports the overall errors for the sum of the population in each of the counties. Here we can see that for the median county, the CCD and CCD/CCR models produce similar APEs but the CCD/CCR model tends to produce slightly lower APEs when compared to the purely CCD model. In all cases, the errors associated with the CCR model are greater than the CCD or CCD/CCR varieties. The ALPEs for all three methods are also relatively low, with CCD model producing the lowest bias. All three methods produce positive bias, suggesting the models are likely to overproject.

**Figure 3:**
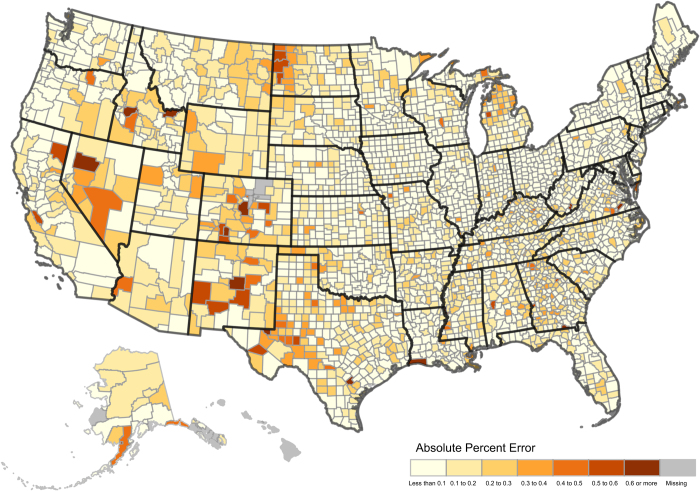
Map of county errors of the total population in 2015 using the CCD/CCR model. This figure shows the geographic distribution of absolute percent errors. Most states and counties have low error rates of the total population with isolated pockets of large errors. The missing counties in Colorado are due to geographic boundary changes associated with the creation of Broomfield County in 2001.

Figure 3 shows the absolute percent errors associated with the total population for the CCD/CCR model in U.S. counties in 2015. Most states and counties see relatively low errors with the median APE of just 7.7% by 2015, however some isolated pockets of high errors do exist randomly distributed throughout the United States, specifically in the Western half of the United States in states such as Colorado and New Mexico.

### Age Structure Error

Table 4 reports the overall errors for age groups at the county level. All three models produce similar APEs. For any given county, the median error is approximately 11% for 2015 with the blended CCD/CCR model producing the lowest errors. Similar to the overall errors, the bias tends to be for over-projection of age groups as all of the ALPEs are positive.

**Figure 4:**
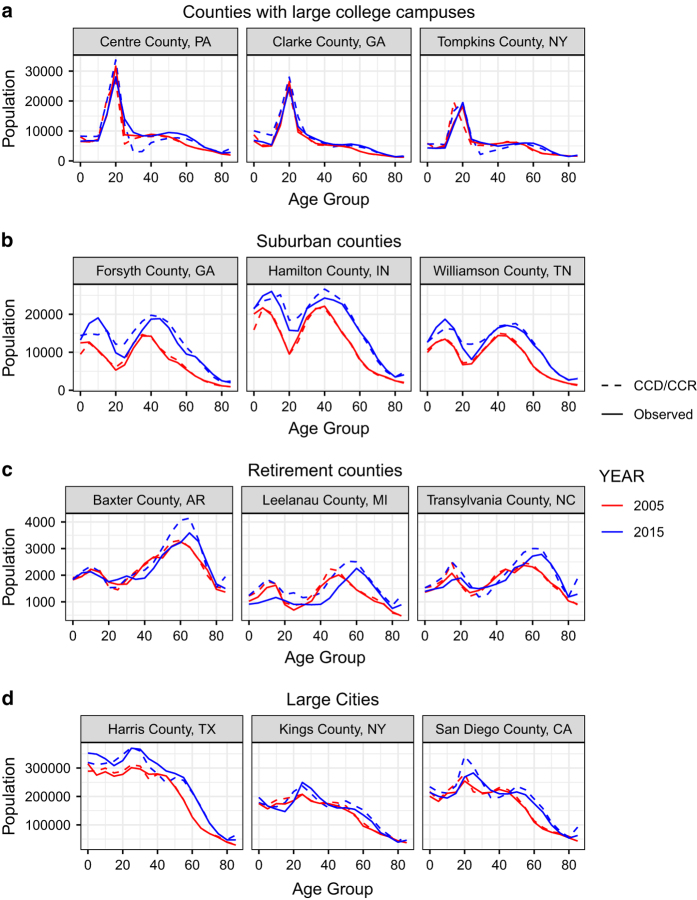
Age structures of various county types. This figure compares the projected age structures to the observed age structures in twelve counties across four county types using the CCD/CCR model. (**a**) Demonstrates counties with major universities, (**b**) demonstrates sample suburban counties, (**c**) demonstrates sample retirement counties, and (**d**) demonstrates sample counties with large cities. All four county types have age structures largely preserved despite widely different age structures.

Figure 4 shows projected age structures in twelve sample counties across four county types – college counties, suburban counties, retirement counties, and large cities. In all four county types the age structures are mostly preserved in the projections. All four county types exhibit differing age structures with important considerations. For college counties, the college-age population (those aged 15–24) do not age in place within those communities. The large population peaks in those counties show great in-migration at the college ages and then great out-migration afterwards. In suburban counties, a “double hump” age structure is typically present with large numbers of both adolescents and middle-aged adults. Most twenty-somethings cannot afford to live in affluent suburban areas, move away for school or work, or do not have the family reasons for living there. The large numbers of populations over the age of 55 often identifies retirement communities. Large cities typically contain large numbers of young professionals with few children. The CCD/CCR model is able to mostly reproduce the population dynamics present in these four archetype communities.

**Figure 5:**
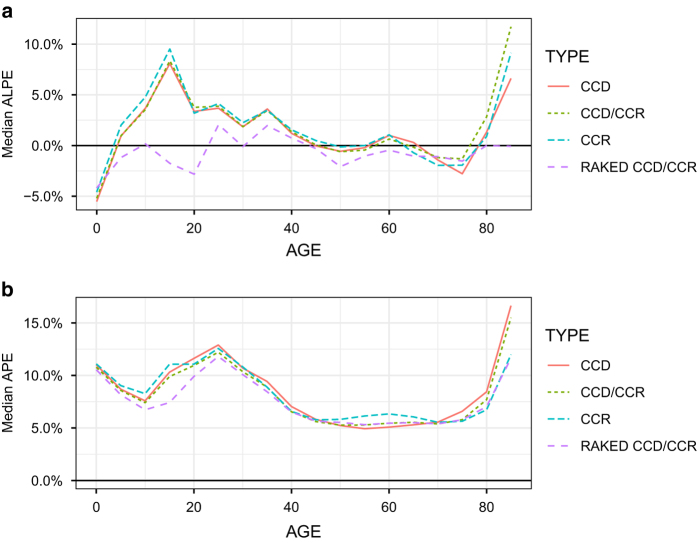
Errors by age group. This figure plots the Median Algebraic Percent Error (ALPE) by age group (**a**) and the Mean Absolute Percent Error by age group (**b**).

Figure 5 shows the Algebraic Percent Errors and Absolute Percent Errors by age group for all three evaluation methods. Three age groups tend to have the greatest bias – 0–4 (~−5%), 15–19 and 85 + (~+ 10%, respectively). Thus, the projections are likely to overproject the number of 15–19 year olds and those aged 85+ and under project the number of 0–4 year olds. The bias in the projections (measured as the ALPE, Fig. 5a) is greatly reduced in nearly all age groups when controlling the populations to the age/sex total of the United States (“RAKE CCD/CCR”). While controlling the projections greatly reduces the bias it does not greatly reduce the overall error rates across age, as measured by APE (Fig. 5b).

### Race Errors

**Figure 6:**
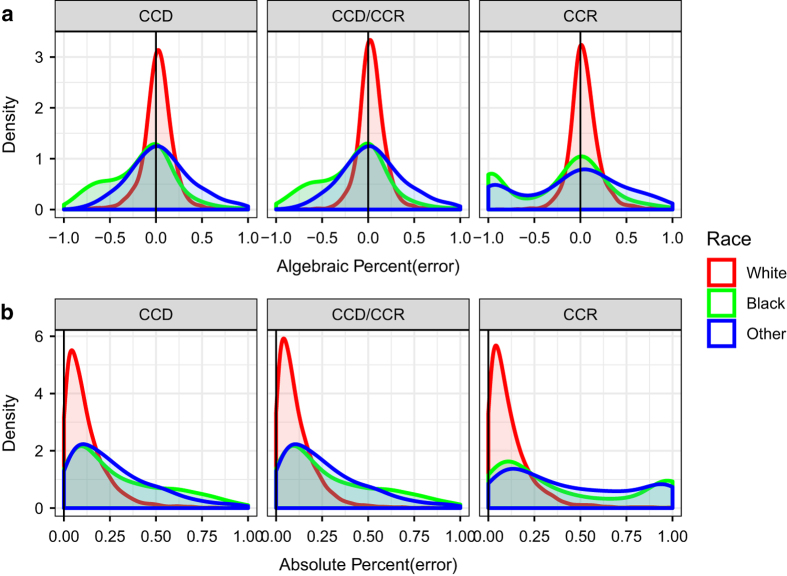
Race group errors. (**a**) Shows the Algebraic Percent Errors for all three methods and (**b**) shows the APE distribution of errors.

Figure 6 reports the ALPE and the APE distribution by race group for all counties. The White race group tends to have the lowest errors associated with the projections, followed by Black, and then Other. This is likely due to the relative population sizes within each race group. Black and Other populations tend to be located in more isolated pockets due to the effects of both institutional and self-assortive segregation from the White population leading to many counties with very small Black and Other populations.

### Projections

**Figure 7:**
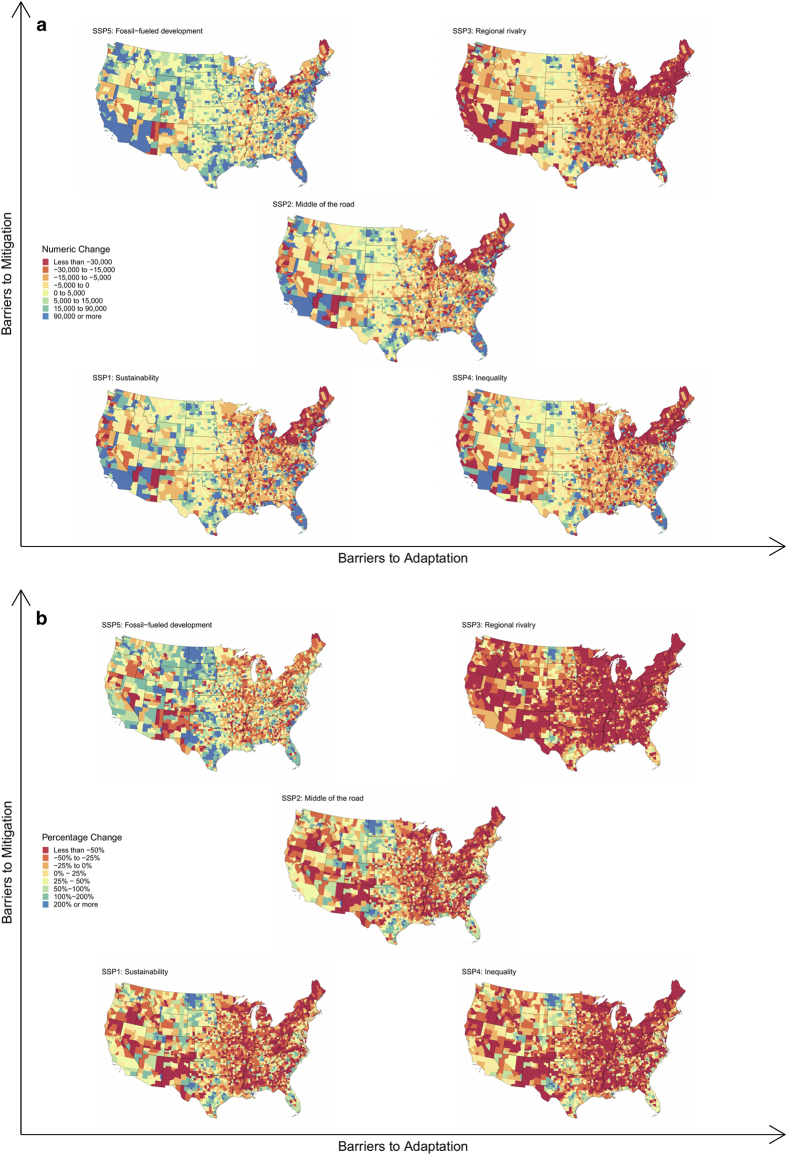
Projected numeric and percentage population changes for the five SSPs between 2020 and 2100 for counties in the continental United States. AK and HI are available in the final projections but are excluded from these maps due to space considerations and to improve interpretability. (**a**) Shows the projected numeric change and (**b**) shows the projected percentage change.

Figure 7 shows county-level numeric and percentage population change for the period 2020–2100 under all five SSPs. The five SSPs lead to substantial differences in geographic growth patterns. For instance, most of California is projected to see increases in population in four of the five SSPs; only SSP3: Regional Rivalry shows projected population declines in southern California. Conversely, the heavily populated North East is projected to see significant population declines in all SSPs except SSP5: Fossil-fueled development. The five SSPs represent different pathways by which the United States could be expected to grow this century.

**Figure 8:**
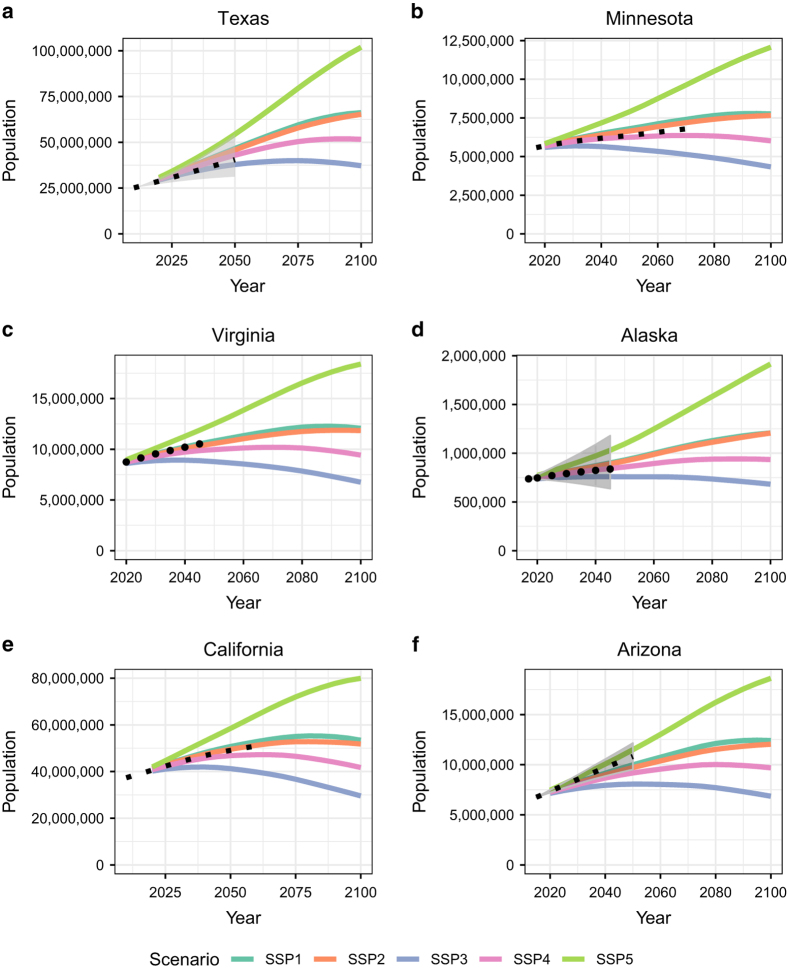
Comparisons to various State-level Population Projections. Several states produce timely population projections. This figure compares six states’ independent population projections to mine produced here. All state-level projections are the black dotted lines. Texas, Alaska, and Arizona include projections of uncertainty and their uncertainty (high, medium, low scenarios) is displayed as the gray shaded area on the respective panels.

Figure 8 shows comparisons to six state-level population projections. These projections are produced by (a) the Texas Demographic Center produced in 2014 (http://txsdc.utsa.edu/data/TPEPP/Projections/Index), (b) the Minnesota State Demographic Center produced in 2015 (https://mn.gov/admin/demography/data-by-topic/population-data/our-projections/), (c) The Weldon Cooper Center for Public Service produced in 2016 (https://demographics.coopercenter.org/virginia-population-projections), (d) the Alaska Department of Labor and Workforce Development produced in 2018 (http://live.laborstats.alaska.gov/pop/projections.cfm), (e) the California Department of Finance produced in 2017 and updated in 2018 (http://www.dof.ca.gov/Forecasting/Demographics/Projections/), and (f) the Arizona Office of Economic Opportunity produced in 2016 (https://population.az.gov/population-projections). These independent state projections utilize different assumptions, methodologies, launch-years, projection-horizons, etc. Texas, Alaska, and Arizona incoporate uncertainty in their projections via deterministic scenarios that incorporate “high”, “medium”, and “low” components of change. For Alaska and Texas, this corresponds to varying migration scenarios and for Arizona this involves varying scenarios of fertility, mortality, and migration. My projections show good agreement with the state-level projections.

## Usage Notes

The dataset generated here provides detailed county-level population projections by age, sex, and race for US counties for the period 2020–2100 that are controlled to the SSPs. Producing high-quality, highly-detailed population projections is a challenging endeavor. With such a large need for sub-national projections and to better understand the changing demographics of the U.S. population, I produced a set of quality, detailed projections and make both the **R** code and subsequent projections available for dissemination to a wide audience. Here, I presented age-sex-race specific population projections for all U.S. counties, an ex-post facto evaluation of the projection methodology, and details on the calculations of these projections.

To ensure quality projections, I employed the use of ex-post-facto evaluations of the projection accuracy for three variant models: purely additive with CCDs, purely multiplicative with CCRs, and a blended model with CCDs in areas projected to grow and CCRs in areas projected to decline. I report the accuracy, bias, and uncertainties associated with these variants using absolute percent error and algebraic percent error.

Overall, the errors reported here are on par with or better than many cohort-component population projection models^18–22^. Table 5 summarizes several population projection evaluations.

While the ex-post-facto evaluation showed relatively low errors, but some areas in the United States, some demographic sub-groups, and some age groups could exhibit greater error rates. These groups include but are not limited to non-white populations, young children under the age of 5, young adults aged 15–19, older adults over the age of 85, and parts of Western US (Idaho, Nevada, New Mexico, and North Dakota, in particular).

These projections, like all projections, involve the use of assumptions about future events that may or may not occur. Users of these projections should be aware that although the projections have been prepared with the use of standard methodologies, documentation of their creation, open-source computer code, and extensive evaluations of their accuracy and uncertainty, they might not accurately project the future population of a state, county, age, sex, or race group. The projections are based on historical trends and current estimates. Any small error in the projections early in the projection horizon could cascade into considerable errors decades later in the projection. Caveat emptor – users beware. These projections should be used only with full awareness of the inherent limitations of population projections in general and with knowledge of the procedures and assumptions described in this document.

## Additional information

**How to cite this article**: Hauer, M. E. Population projections for U.S. counties by age, sex, and race controlled to the Shared Socioeconomic Pathways. *Sci. Data*. 6:190005 https://doi.org/10.1038/sdata.2019.5 (2019).

**Publisher’s note**: Springer Nature remains neutral with regard to jurisdictional claims in published maps and institutional affiliations.

## Supplementary Material



Supplementary Information

## Figures and Tables

**Table 1:** Evaluation of Age/Sex/Race/County joint Errors.

TYPE	num	EVAL	2005	2010	2015
CCD/CCR	336024	Median SAPE	6.2%	8.6%	11.1%
CCD	336024	Median SAPE	6.3%	8.8%	11.6%
CCR	336024	Median SAPE	6.4%	9.1%	12.8%

**Table 2:** Evaluation of overall total errors for the entire United States.

YEAR	TYPE	POPULATION	PRED	% ERROR
2005	CCD	292,540,441	295,278,936	0.9%
	CCD/CCR	292,540,441	295,648,069	1.1%
	CCR	292,540,441	297,006,438	1.5%
2010	CCD	306,383,005	311,439,453	1.7%
	CCD/CCR	306,383,005	312,185,612	1.9%
	CCR	306,383,005	318,289,491	3.9%
2015	CCD	317,731,270	327,977,760	3.2%
	CCD/CCR	317,731,270	329,078,676	3.6%
	CCR	317,731,270	359,037,997	13.0%

**Table 3:** Evaluation of overall errors for each county.

TYPE	n	EVAL	2005	2010	2015
CCD/CCR	3127	Median APE	2.4%	4.8%	7.7%
CCD	3127	Median APE	2.5%	5.0%	8.1%
CCR	3127	Median APE	2.5%	5.2%	8.9%
CCD	3127	Median ALPE	0.8%	0.7%	2.9%
CCD/CCR	3127	Median ALPE	1.0%	1.2%	3.3%
CCR	3127	Median ALPE	1.1%	1.9%	4.6%

**Table 4:** Evaluation of Age Group Errors.

TYPE	n	EVAL	2005	2010	2015
CCD/CCR	56286	Median APE	5.3%	8.0%	10.8%
CCD	56286	Median APE	5.3%	8.2%	11.4%
CCR	56286	Median APE	5.4%	8.2%	11.3%
CCD	56286	Median ALPE	0.7%	0.6%	2.3%
CCD/CCR	56286	Median ALPE	0.9%	1.0%	2.8%
CCR	56286	Median ALPE	1.1%	1.1%	2.6%

**Table 5:** Comparable Population Projection Errors.

Authors	Location	Methods	Analysis	Metric	Projection Horizon	Errors
Wilson^19^	New South Wales	Ten cohort-component and CCR variants	Total population	Median APE	10-years	3.6%–6.5%
Rayer^21^	US counties	Seven extrapolation approaches	Total population	Mean APE	10-years	9.3%–13.7%
Smith & Tayman^20^	US counties	Cohort-component	Age Structure	Mean APE	10-years	6.7%–10.6%
Smith & Tayman^20^	Florida counties	CCRs/Cohort-component	Age Structure	Mean APE	10-years	4.9%–15.4%
Sprague^18^	US Counties	CCRs	Age structure	Mean APE	10-years	6%–16%
Raftery *et al.*^22^	Countries	Bayesian Cohort-Component	Total population	Mean APE	20-years	2.7%
