# Supplementary material for: Population projections for U.S. counties by age, sex, and race controlled to shared socioeconomic pathway
Source: Sci Data. 2019 Feb 5;6:190005. doi: 10.1038/sdata.2019.5 (PMC6362894; doi:10.1038/sdata.2019.5)
Supplement: Supplementary Information [file sdata20195-s2.pdf]

Supplementary Information

The final population projections are controlled to the Shared Socioeconomic Pathways. However, it might be informative to see the results of the uncontrolled population projection prior to raking to the SSPs. Supplementary Figure 1 shows the results of the uncontrolled population projections for 2020-2100 compared to the five SSPs. Between 2020 and approximately 2050, the uncontrolled population projections compare very favorably to SSP1 and SSP2 (Sustainability and Middle of the Road, respectively). However, the latter-half of the 21st Century sees an uncontrolled US total approximately 35% higher than SSP2.

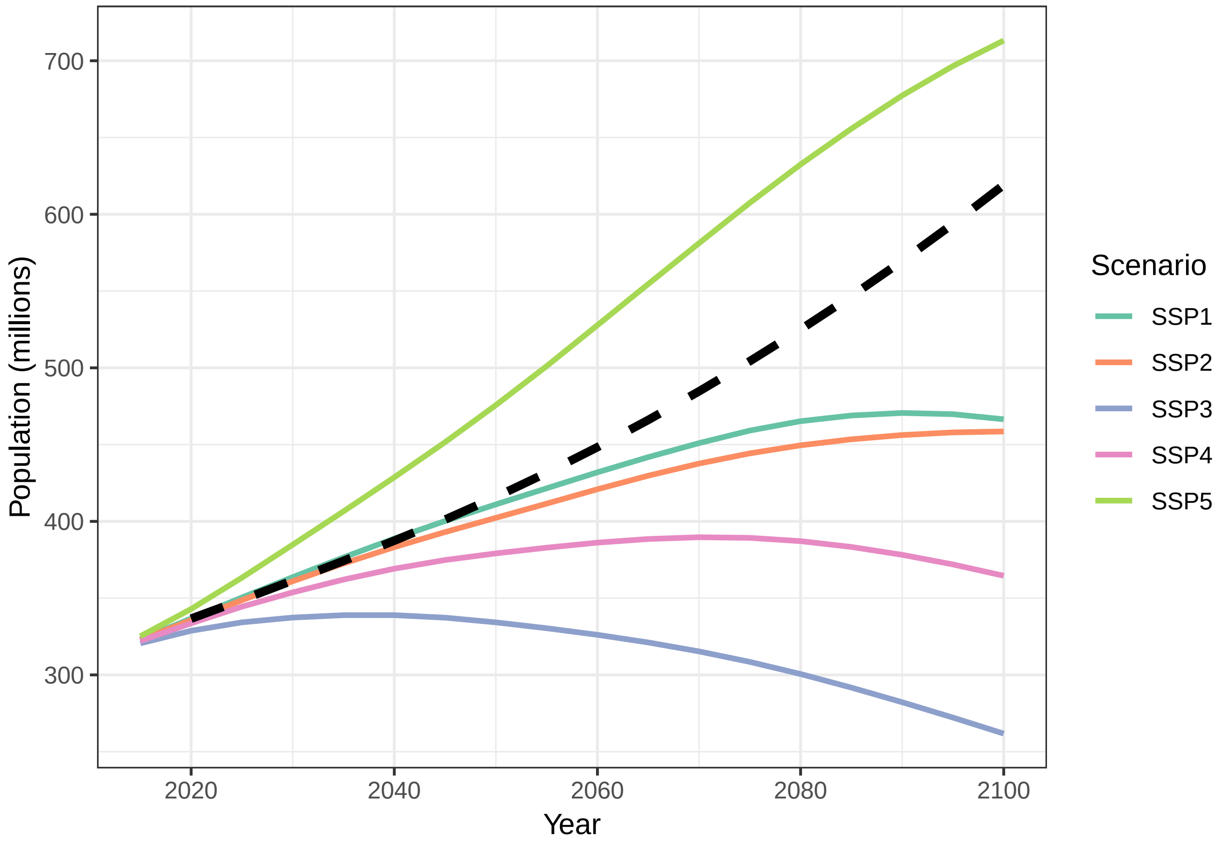

Supplementary Figure 1: **Uncontrolled Population Projection Comparison.** The uncontrolled population projection is the dashed black line.
